# Analysis of Factors Influencing Air Quality in Different Periods during COVID-19: A Case Study of Tangshan, China

**DOI:** 10.3390/ijerph20054199

**Published:** 2023-02-26

**Authors:** Wen-Lu Wu, Chun-Yan Shan, Jing Liu, Jing-Lin Zhao, Jin-Yun Long

**Affiliations:** College of Environmental Science and Engineering, Nankai University, Tianjin 300350, China

**Keywords:** air quality, COVID-19, anthropogenic factors, meteorological factors, difference-in-differences analysis, backward trajectory cluster

## Abstract

This study aimed to analyze the main factors influencing air quality in Tangshan during COVID-19, covering three different periods: the COVID-19 period, the Level I response period, and the Spring Festival period. Comparative analysis and the difference-in-differences (DID) method were used to explore differences in air quality between different stages of the epidemic and different years. During the COVID-19 period, the air quality index (AQI) and the concentrations of six conventional air pollutants (PM_2.5_, PM_10_, SO_2_, NO_2_, CO, and O_3_-8h) decreased significantly compared to 2017–2019. For the Level I response period, the reduction in AQI caused by COVID-19 control measures were 29.07%, 31.43%, and 20.04% in February, March, and April of 2020, respectively. During the Spring Festival, the concentrations of the six pollutants were significantly higher than those in 2019 and 2021, which may be related to heavy pollution events caused by unfavorable meteorological conditions and regional transport. As for the further improvement in air quality, it is necessary to take strict measures to prevent and control air pollution while paying attention to meteorological factors.

## 1. Introduction

Starting from the first day of lockdown declared in Wuhan on 23 January 2020, regions in China where the coronavirus disease 2019 (COVID-19) was found entered the Level I response period of the epidemic. A series of measures were implemented to limit anthropogenic activities, such as isolation, community containment, suspension of work and production, and the blocking of traffic arteries [1]. As the outbreak receded, the level of epidemic prevention and control in various places was lowered [2].

Related research has generally concluded that the COVID-19 control measures implemented in China improved air quality in the short term, and most research focused on the Level I response period and the Spring Festival period. Compared with 2019, the national average PM_2.5_, PM_10_, SO_2_, NO_2_, and CO concentrations decreased significantly during the period from 24 January to 30 April 2020, and the O_3_ concentration did not change significantly or showed an upward trend in some areas [3,4,5]. With the resumption of work and production at the end of February, PM_2.5_ and NO_2_ concentrations in most cities began to increase gradually [6,7]. Compared with the previous Spring Festival holiday and the days before the Spring Festival, the anthropogenic emissions of PM_2.5_, SO_2_, and NO_2_ during the Spring Festival in 2020 were significantly reduced, and the concentration of pollutants in most cities was significantly reduced [8,9]. Furthermore, the long-term impact of COVID-19 control measures on air quality is one future research direction [10]. Li et al. [11] assessed the effect of different emergency response levels on air quality in the Guangdong–Hong Kong–Macao Greater Bay Area (GBA), and found that the concentration of PM_2.5_, PM_10_, NO_2_, CO, and O_3_ were not significantly different during the Level II and III response periods. Tomasz Turek et al. [12] demonstrated that restricting anthropogenic activities did not have an impact on long-term changes and trends in air quality in Wroclaw, Poland. In contrast, Elçin Tan [13] found that the average air quality in Istanbul improved by about 17% during the long-term lockdown period (12 March 2020 to 30 June 2021). Overall, whether it is a short-term or long-term impact, the degree of change in air quality varied from region to region during COVID-19 [14,15].

The heavy pollution events in China are typically associated with local emissions, regional transport, enhanced secondary production, and unfavorable meteorology [16,17]. During COVID-19, the North China Plain was still disturbed by weather such as smog and sandstorms, and heavy pollution events occurred frequently [18]. The Beijing–Tianjin–Hebei (BTH) region still experienced high concentrations of PM_2.5_ due to unfavorable meteorological conditions [19]. Almost all studies affirm the positive effect of reducing anthropogenic emissions on air quality including some areas where adverse meteorological conditions offset the effects of anthropogenic emission reductions, especially in heavy industrial cities in northern China [20].

Machine learning models are often used to quantitatively analyze the impact of anthropogenic emissions and meteorological factors on air quality [21,22]. Linear regression models are one of the most popular machine learning models, which have worked well simulating pollutant concentrations during the pandemic. Compared to Random Forest (RF) models and K-Nearest Neighbors (KNN) models, Multiple Linear Regression (MLR) had the best overall performance of all indicators when predicting air pollutant concentrations for urban traffic sites in Spain using meteorological variables as independent variables [23]. MLR, 3D models, and Artificial Neural Network (ANN) models were used to simulate PM_10_ concentrations before and during confinement by COVID-19 in South Lima, and MLR had the highest correlation coefficient (R^2^ = 0.6166) [24].

Most existing studies on China are at the national, provincial, and other large regional level, while studies on cities focus on developed cities such as Beijing and Shanghai, of which the tertiary industry accounts for a much higher proportion than the secondary industry. Studies on common cities dominated by the secondary industry are rarely seen. This study chose Tangshan in Hebei Province as a case to reflect the impact of the epidemic on industrial cities in northern China. The study was carried out on three time scales, including the Spring Festival, the short-term phase of the Level I response, and the long-term phase including the Level I, Level II, and Level III response, so as to distinguish the dominated factors in the different periods. The comparative analysis and difference-in-differences (DID) model were used to analyze the variation in the air quality index (AQI) and six pollutants (i.e., PM_2.5_, PM_10_, SO_2_, NO_2_, CO, and O_3_). An MLR model, a backward trajectory model, a cluster analysis method, and a potential source contribution function (PSCF) analysis method were used to investigate the main factors affecting air quality.

## 2. Data and Methods

### 2.1. Study Area

**Figure 1:**
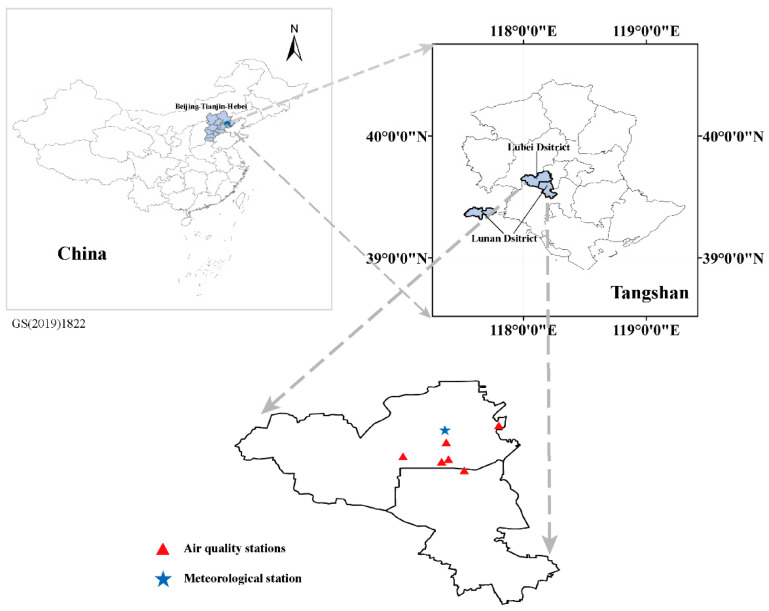
Location of Tangshan and distribution of meteorological and air quality stations.

Tangshan is located in the east of Hebei Province, bordering Beijing and Tianjin on the west (Figure 1). Its economic strength was stronger among northern cities since Tangshan’s GDP ranked first in Hebei Province in 2021. Tangshan is a typical heavy industry city with its secondary industry GDP accounting for 55.24% of the total GDP (http://www.tangshan.gov.cn/zhuzhan/jjsj, accessed on 31 January 2023). The main industries are heavy industry and resource mining industry, while steel, coal, cement, and porcelain are the four major industries. Its steel production accounted for 12.05% of the total steel production in China in 2021 (https://data.stats.gov.cn, accessed on 31 January 2023).

Tangshan belongs to the BTH region, which is China’s capital economic circle, including Beijing, Tianjin, and 11 cities in Hebei Province. The BTH region is one of China’s most polluted regions, with Tangshan having the worst air quality. Although a series of air pollution control measures have been implemented in recent years, heavy pollution events are still inevitable, especially during the winter season, in which buildings require heating [25].

### 2.2. Time Periods

The data analyzed in the article covered the period from 24 January to 30 June, in 2017 to 2021. A comparative analysis was conducted on the data of these five years, in which 2017–2019 are the years without the epidemic, and 2020 and 2021 are the first and second year of the epidemic, respectively.

**Figure 2:**
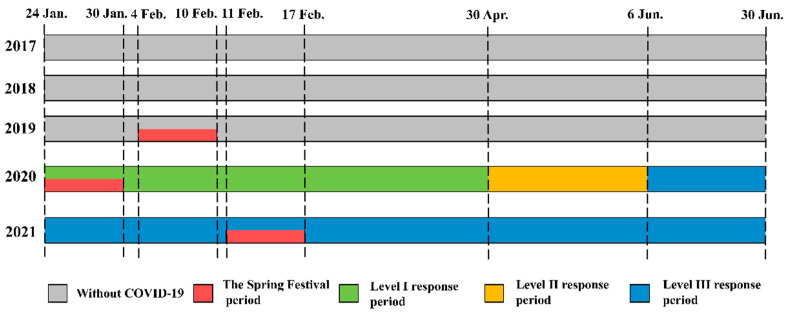
Visualization of the three different periods during COVID-19 analyzed in this study.

The anthropogenic control measures varied widely in different periods of the COVID-19 outbreak. 24 January 2020 was the first day of the Spring Festival and the day in which the Hebei Provincial Government activated the Level I response to major public health emergencies. At the beginning of the response in Tangshan, public transportation was suspended across the city, fireworks and firecrackers were banned, and all villages, communities, and enterprises were placed under close management. After the response level was adjusted to Level II on 30 April 2020, the epidemic control status changed from wartime to normal, but measures such as access control and strict vehicle control were still in effect. After the response level was adjusted to Level III on 6 June 2020, normal working conditions and daily life gradually resumed, but basic measures such as wearing masks, not gathering, and reducing travel were still being implemented. Due to the normalization stage of the epidemic prevention and control, the intensity of control measures in 2021 may have been weaker than that in 2020. Therefore, the analysis conducted in this study was divided into the following three periods based on the occurrence of COVID-19 and the level of response to it (Figure 2).

*COVID-19 period: 24 January to 30 June.* This period includes the Level I, II, and III response periods in 2020. The comparison with the same period from 2017 to 2019 and 2021 aimed to analyze the long-term impact of COVID-19 control measures on air quality.

*Level I response period: 24 January to 30 April.* As the COVID-19 control measures during the Level I response were significantly stricter than those during the Level II and III response periods in 2020, this period was selected to investigate the short-term impact of COVID-19 control measures on air quality.

*The Spring Festival period.* The Spring Festival is a traditional Chinese holiday period that lasts for 7 days. During the Spring Festival that was not affected by the epidemic, production activities reduced and population movements increased. During the Spring Festival during the epidemic, anthropogenic activities were relatively reduced. Therefore, a separate interannual comparative analysis was conducted for the Spring Festival period from 2019 to 2021.

### 2.3. Data Sources

The data of AQI and concentrations for six conventional atmospheric pollutants (PM_2.5_, PM_10_, SO_2_, NO_2_, CO, and O_3_-8h) from six air quality monitoring stations in the downtown area of Tangshan were obtained from the comprehensive stereo observation data sharing platform for the Beijing–Tianjin–Hebei area and its surrounding regions (http://123.127.175.60:8765/siteui/index, accessed on 31 January 2023).

Among the above-mentioned data, O_3_-8h corresponded to the average value of the maximum ozone concentration over an 8 h period each day. The hourly value was the average of hourly observations for the six air quality monitoring stations. The AQI is a dimensionless index that can quantitatively describe air quality, and it is calculated based on the concentrations of the six conventional atmospheric pollutants mentioned above in China [26]. The index defines six levels of air quality: excellent (0 < AQI ≤ 50), good (51 < AQI ≤ 100), lightly polluted (101 < AQI ≤ 150), moderately polluted (151 < AQI ≤ 200), heavily polluted (201 < AQI ≤ 300), and severely polluted (AQI > 300). When the AQI is >50, the pollutant with the largest individual air quality index (IAQI) is considered as the primary pollutant.

The data of near-ground meteorological parameters (temperature, humidity, wind speed, atmospheric pressure, visibility, and precipitation) were obtained from the Huiju Data platform (http://hz.hjhj-e.com/home, accessed on 31 January 2023), while the economic data were acquired from the website of Tangshan Bureau of Statistics (http://new.tangshan.gov.cn, accessed on 31 January 2023).

The data on air mass trajectories were sourced at the National Center for Environmental Prediction (NCEP, http://ftp://arlftp.arlhq.noaa.gov/pub/archives/gdas1, accessed on 31 January 2023), and were generated every 6 h.

### 2.4. Difference-In-Differences (DID) Model

The DID model is a powerful tool in the evaluation of policy effects [27]. The differences in air quality can be attributed to either response measures or seasonal influences or both. The DID model was employed to measure whether the response measures were effective. It was assumed that air quality in the same period in different years has similar trends [28]. Therefore, data from 2017 to 2019 and 2021 were used as the control group, while data from 2020 were used as the experimental group. The DID model setting was as follows:

(1) $$Y_{it}=\alpha Measure_{it}\times Post_{it}+\beta Y_{it-1}+\gamma X_{it}+\delta \left(Trend_{i}\right)+\varepsilon_{it}$$

where $Y_{it}$ is the dependent variable, which represents the air quality of Tangshan on t day in i year, denoted by AQI. Measure is a dummy variable that represents whether Tangshan launched a response to the public health emergency. If launched, Measure=1; otherwise, Measure=0. Post is a dummy variable that reflects whether day t is before or after the day node (January 24), and before the day node, Post=0; otherwise, Post=1. In addition, considering that the air quality on a given day is affected by that of the previous day and meteorological factors, the explanatory variables $Y_{it-1}$ and $X_{it}$ were added to the model. Meteorological factors include temperature, humidity, wind speed, visibility, and precipitation. It is generally believed that air quality has a trend of improving year by year in China [29], which we assume also exists in Tangshan. The model was controlled with a dummy variable Trend, which was introduced only when the number of study days per year exceeded six months.

It is a necessary condition for the application of the DID model to pass the parallel trend test: the seasonal influences of the experimental and control groups must be similar without the COVID-19 control measures [30].

### 2.5. Multiple Linear Regression (MLR) Model

Regression analysis is generally used to statistically describe the relationship between independent and dependent variables, and the value of the latter can be estimated based on that of the former [31]. When independent variables are related to a dependent variable, a predictive MLR equation can be established [32], which is expressed as:

(2) $$Y=\beta_{0}+\beta_{1}X_{1}+\beta_{2}X_{2}+\cdots +\beta_{k}X_{k}+\varepsilon$$

where Y is the dependent variable; $X_{1},X_{2}\cdots ,X_{k}$ are independent variables; $\beta_{0},\beta_{1}\cdots ,\beta_{k}$ are linear regression parameters; and *ε* is a random error term that follows a normal distribution.

### 2.6. Backward Trajectory Cluster Analysis and PSCF Analysis

Backward trajectory cluster analysis and potential source contribution function (PSCF) analysis were conducted using MeteoInfo software [33] and TrajStat plug-in [34].

The Hybrid Single-Particle Lagrangian Integrated Trajectory (HYSPLIT) model was employed to obtain the backward trajectories. The HYSPLIT model, which has been jointly developed by the Air Resources Laboratory of the National Oceanic and Atmospheric Administration (NOAA) and the Australian Meteorological Administration to simulate the trajectory of air masses, is widely used in the fields of meteorology and atmospheric sciences. Cluster analysis was conducted as a method to group air mass trajectories with similar geographical origins, and PSCF analysis was carried out to qualitatively identify potential pollution sources based on conditional probability functions [35]. Based on spatial grid calculations, the research area selected in this study was divided into i×j uniform grids; the ratio of the number of polluted air trajectory endpoints $m_{ij}$ through a certain grid (i,j) to the total air trajectory endpoint number $n_{ij}$ of the grid was defined as the PSCF value ($P_{ij}$). To eliminate the effect on the results caused by the overestimation of some grids with a very small number of trajectories, a weighting factor ($W_{i}$) was introduced [36]:

(3) $$W_{i}=\{\begin{array}{l} 1.00\;\;\;\;\;\;\;\;\;\;\;\;\;\;\;\;\;\;\;\;\;\;\;n_{ij}\geq 4W_{ij}\\ 0.70\;\;\;\;\;\;\;\;\;\;\;\;W_{ij}\leq n_{ij}<4W_{ij}\\ 0.42\;\;\;\;\;\;\;0.5\;W_{ij}\leq n_{ij}<W_{ij}\\ 0.05\;\;\;\;\;\;\;\;\;\;\;\;\;\;\;\;\;\;\;\;\;\;\;n_{ij}<0.5\;W_{ij} \end{array}$$

where $n_{ij}$ represents the total residence time of all trajectories in the grid (i,j), and $W_{ij}$ represents the average number of trajectories passed by each grid.

The Materials Bureau air quality station (E 118.17°, N 39.63°) was selected as the receiving point, and the starting height of the trajectory simulation was set at 500 m. The air mass trajectory was calculated backward for 24 h each time.

## 3. Results and Discussion

### 3.1. Analysis of Factors Influencing Air Quality during COVID-19

#### 3.1.1. Interannual Differences in Air Quality

The average AQI value and concentration of the six pollutants observed in different periods are shown in Supplementary Materials Table S1. From 24 January to 30 June 2020, the average AQI value in Tangshan was 93.20, which corresponded to the second air quality level (good), and the average concentrations of PM_2.5_, PM_10_, SO_2_, NO_2_, CO, and O_3_-8h were 50.03 µg/m^3^, 93.04 µg/m^3^, 19.86 µg/m^3^, 41.36 µg/m^3^, 1.25 mg/m^3^, and 110.17 µg/m^3^, respectively, which were all below the Ambient Air Quality Standard Grade II (PM_2.5_ 75 µg/m^3^, PM_10_ 150 µg/m^3^, SO_2_ 150 µg/m^3^, NO_2_ 80 µg/m^3^, CO 4 mg/m^3^, O_3_-8h 160 µg/m^3^).

Compared with the average data of the same period from 2017 to 2019, the daily average concentration of pollutants from 24 January to 30 June in 2020 decreased to varying degrees, with SO_2_ and O_3_-8h showing the largest (42.78%) and smallest (6.13%) reductions, respectively. There were significant differences in the concentrations of PM_2.5_ at the 95% confidence interval between 2017–2019 and 2020, and significant differences in the concentrations of PM_10_, SO_2_, NO_2_, and CO at the 99% confidence interval. There being no significant decrease in O_3_ in Tangshan was mainly attributed to the enhanced atmospheric oxidation capacity [37]. During the same period in 2021, the concentrations of PM_2.5_, PM_10_, and NO_2_ increased slightly compared with those observed in 2020, potentially meaning that the Level I and II response had a greater impact on air quality than the Level III response. SO_2_ and CO values in 2021 were lower than those in 2020; this was mainly related to the continuous implementation of the “coal to gas” and “coal to electricity” policies [38].

**Figure 3:**
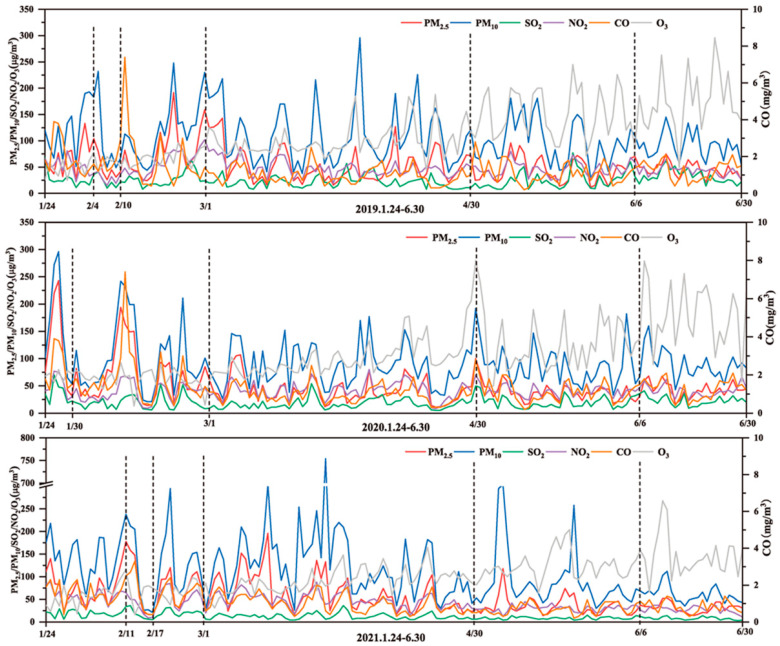
Variation in the daily average concentrations of pollutants from 24 January to 30 June for the 2019–2021 period.

The interannual variability of six atmospheric pollutant concentrations was compared from 2019 to 2021 (Figure 3). PM_2.5_, PM_10_, SO_2_, NO_2_, and CO concentrations showed similar variation patterns, suggesting that they might have common sources and influencing factors. O_3_ is a secondary pollutant affected by the combined action of nitrogen oxides (NOx) and volatile organic compounds (VOCs) [39,40], and due to the complexity of its mechanism of generation, it shows a different pattern of variation compared to the other pollutants. Overall, the range of variation in the concentration of each pollutant in 2020 was smaller than that in 2019 and 2021, and the number of clean air days was significantly higher. The proportion of days in which PM_2.5_ concentration exceeded the standard (75 µg/m^3^) in 2019, 2020, and 2021 was 19.2%, 13.29%, and 25.95%, respectively, indicating that the health risks associated with the exposure to particulate matter were clearly lower in 2020 [41].

#### 3.1.2. Analysis of Significant Difference Based on DID Model

The long-term effect of COVID-19 control measures on the air quality from 1 January to 30 June in 2020 was explored by conducting a DID model. The descriptive statistical results of the main variables and the estimation result of the model are listed in Supplementary Materials Table S2A and Table S3A, respectively. The coefficient α was found statistically significantly negative at the 1% level, which indicated that air quality improved significantly during COVID-19 in Tangshan. The coefficient δ was significantly negative at the 1% level, which confirmed the previous hypothesis, i.e., Tangshan’s air quality shows a trend of improvement year by year.

However, the result of the parallel trend test opposed the use of the DID model (Supplementary Materials Figure S1A). In 2017–2019 and 2021, the coefficients were not significantly different from 2020, which cannot demonstrate that the control measures had a significant long-term impact on air quality.

#### 3.1.3. Impact of Meteorological Factors

**Figure 4:**
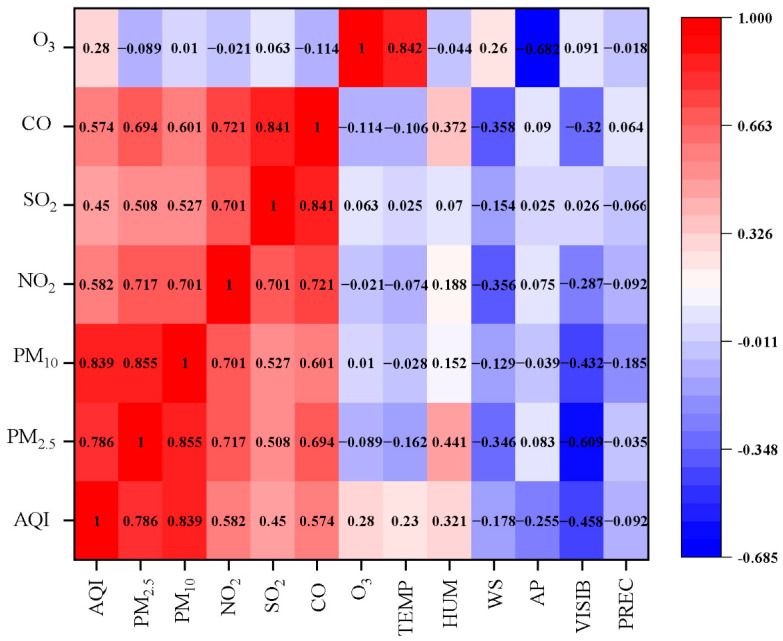
Heat map of the Spearman correlation coefficient for AQI, pollutants, and meteorological parameters. Abbreviations: TEMP, temperature; HUM, humidity; WS, wind speed; AP, atmospheric pressure; VISIB, visibility; PREC, precipitation. Significant correlation was set at *p* < 0.05.

Meteorological factors have a strong influence on the variation in air pollution levels [42,43]. To explore the influence of meteorological factors on air quality, the Spearman correlation analysis was conducted among AQI, six pollutants, and six meteorological parameters using the SPSS software. The data from 24 January to 30 June, 2017–2021, were analyzed since a longer time series could reduce the impact of uncertainties. The results are shown in Figure 4: temperature was strongly positively correlated with O_3_ (*r_s_* = 0.842, *p* < 0.01), and negatively correlated with PM_2.5_, PM_10_, NO_2_, and CO; humidity was positively correlated with pollutants other than O_3_, and moderately positively correlated with PM_2.5_ and CO (*r_s_* = 0.441, *p* < 0.01; *r_s_* = 0.372, *p* < 0.01); wind speed was negatively correlated with pollutants other than O_3_, as wind could transfer, diffuse, and dilute pollution; atmospheric pressure was strongly negatively correlated with O_3_ (*r_s_* = −0.682, *p* < 0.01), and was positively correlated with PM_2.5_, NO_2_, SO_2_, and CO; visibility was negatively correlated with PM_2.5_, PM_10_, NO_2_, and CO, and was more significantly correlated with particulate matter; precipitation was negatively correlated with pollutants other than CO, but the overall correlation was low.

From 24 January to 30 June 2020, the average temperature in Tangshan was 10.56 °C, humidity was 54.30%, wind speed was 1.92 m/s, atmospheric pressure was 1015 hPa, visibility was 16.09 km, and precipitation was 0.73 mm (Supplementary Materials Table S4). These were mostly unfavorable meteorological conditions compared to those recorded in 2017–2019 and 2021, indicating that meteorological factors in 2020 had a negative effect on the improvement in air quality. However, overall, the air quality in 2020 was better than that in 2017–2019 and 2021, showing that anthropogenic factors might played a positive role in improving the air quality and masked the impact of meteorological factors.

#### 3.1.4. Impact of COVID-19 Control Measures

Based on the Air Pollution Source Emission Inventory of Tangshan (2017), it was calculated that industrial processes, dust, fossil fuel combustion, and vehicles were the main sources of conventional air pollutants (Supplementary Materials Figure S2). In particular, the emission contribution rates of industrial processes to PM_2.5_, PM_10_, SO_2_, and CO were 53.55%, 38.03%, 78.16%, and 90.68% in 2017, respectively. The measures adopted to control the epidemic had an impact on the emission intensity of the above-mentioned pollution sources. Therefore, the available economic indicators closely related to them were selected as parameters reflecting the impact of the variation in anthropogenic emissions on air quality during the 2019–2021 COVID-19 period (Table 1).

The GDP of the secondary industry in the first and second quarters of 2020 was significantly lower than that in 2019 and 2021, and the total profit of manufacturing in 2020 was about 50% of that of 2019, which indicates that COVID-19 had a certain impact on industrial production in 2020. However, the product output related to people’s livelihood, such as steel, coal, cement, and power generation, was not significantly different from that reported in 2019—a year not affected by COVID-19—which demonstrates that the drop in air pollutant concentrations was likely due to the closure of industries not related to livelihood [44]. The secondary industry includes industry and construction. The electricity consumption of industry dropped significantly in the first quarter of 2020, and that of construction, which is a major source of particulate matter, showed the same trend. As work and production resumed in late February, the levels of power usage gradually returned to normal. The increase in electricity consumption by urban and rural residents and the apparent decline in the level of transportation in the first quarter of 2020 may be due to the lockdowns and travel controls imposed in this year, which led to a reduction in NO_2_, SO_2_, and CO concentrations. In summary, COVID-19 control measures had a significant impact on air pollutant emissions, which in turn affected pollutant concentrations [45].

### 3.2. Analysis of Factors Influencing Air Quality during the Level I Response

#### 3.2.1. Interannual Differences in Air Quality

Existing research shows that the COVID-19 outbreak improved China’s air quality in the short term [46], and the impact of related measures on pollutant concentrations was gradually weakened as the prevention and control levels were lowered [47]. We conducted an in-depth comparative analysis of AQI and pollutant concentrations to examine the impact of COVID-19 control measures on air quality during the Level I response. The average concentrations of PM_2.5_, PM_10_, SO_2_, NO_2_, CO, and O_3_-8h from 24 January to 30 April 2020 were 56.22 µg/m^3^, 96.93 µg/m^3^, 19.38 µg/m^3^, 41.73 µg/m^3^, 1.32 mg/m^3^, and 87.80 µg/m^3^ (Supplementary Materials Table S1), respectively. Compared with the same period in 2017–2019, the concentrations decreased by 21.26%, 24.58%, 46.02%, 26.08%, 23.70%, and 0.54%, respectively; of these, the variations in PM_2.5_, PM_10_, NO_2_, SO_2_, and CO were significantly different (*p* < 0.01). In the same period in 2021, the concentrations of PM_2.5_, PM_10_, and NO_2_ increased by 19.57%, 38.86%, and 19.39%, respectively, and the differences were significant (*p* < 0.05).

#### 3.2.2. Analysis of Significant Difference Based on DID Model

The DID model was carried out to investigate the impact of measures on air quality during Level I response, with the data from 23 days before and after the Level I response. The descriptive statistical result of the main variables and the estimation result of the model are reported in Supplementary Materials Table S2B and Table S3B, respectively. Similar to the long-term study results, the coefficient α was found statistically significantly negative at the 1% level, which indicates that air quality improved significantly during the Level I response.

The parallel trends test was conducted, and Supplementary Materials Figure S1B presents the estimated coefficients and their 95% confidence intervals. In 2017–2019, the coefficient α was significantly positive, indicating that air quality after January 24 was significantly worse than before. In 2020, the coefficient α changed from significantly positive to significantly negative, which means that 23 days after the Level I response, the air quality suffered an impact. Obviously, this result further supported the use of the DID model.

Additionally, the results of the DID model illustrated that the air quality improving was mainly attributed to anthropogenic factors during the Level I response, while natural factors might have played a counterproductive role. Current environmental protection measures include basic environmental protection measures and indirect COVID-19 control measures, and good air pollution control strategies can provide crucial impacts in reducing air pollution [48].

#### 3.2.3. Impact of COVID-19 Control Measures Based on MLR Model

Assuming that basic environmental protection measures remained unchanged from 2017 to 2021, regression models were established to identify the relationship between meteorological parameters and AQI, and to quantify the impact of COVID-19 control measures on air quality. After the comparative analysis of the MLR model and the principal component regression (PCR) model, the former, which included the meteorological parameters as the independent variable and logAQI as the dependent variable, was finally adopted for subsequent simulations (Supplementary Materials Text S1, Tables S5–S7 and Figures S3 and S4). In studies simulating air quality using meteorological data, the difference between the simulated and observed values can be considered the influence of COVID-19 control measures. Data from 1 February to 30 April, 2017–2019, were used to build MLR models simulating air quality from 1 February to 30 April, 2019–2021.

**Figure 5:**
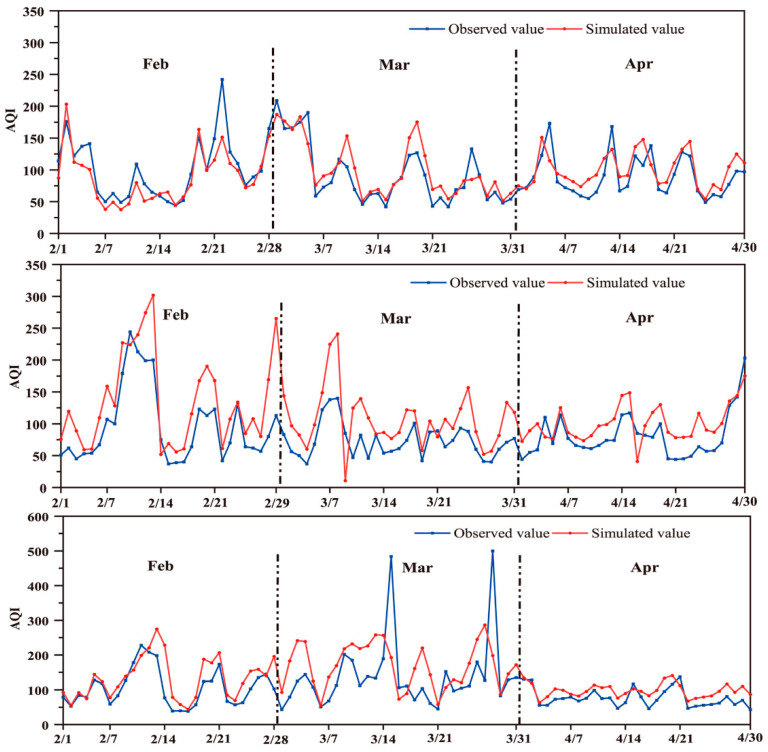
Simulated daily AQI values based on MLR models for February, March, and April 2019–2021.

The model simulation results for February, March, and April 2019–2021 are shown in Figure 5. In 2019 (the year without COVID-19), the simulated monthly average values of AQI for the three months were 88.52 µg/m^3^, 100.23 µg/m^3^, and 99.66 µg/m^3^, respectively, and compared to them, the observed values increased by 12.54% for February, and decreased by 8.83% and 11.73% for March and April, respectively. In 2020, the observed monthly average values in February, March, and April were 96.79 µg/m^3^, 73.19 µg/m^3^, and 80.50 µg/m^3^, respectively, of AQI, while the simulated values were 136.45 µg/m^3^, 106.74 µg/m^3^, and 100.68 µg/m^3^, respectively. Therefore, the reduction in AQI caused by COVID-19 control measures in February, March, and April during the Level I response in 2020 were estimated at approximately 29.07%, 31.43%, and 20.04%, respectively. The reduction in February, March, and April during the Level III response in 2021 were estimated at approximately 20.68%, 18.37% and 22.04%, respectively.

The above results showed that COVID-19 control measures played a role in improving air quality. The impact of anthropogenic factors on the AQI in February and March was significantly higher in 2020 than in 2021, which might be attributable to the difference in the Level I and Level III response. This result also showed that, to a certain extent, air pollution could be reduced by strengthening control efforts. However, the AQI in April was slightly lower in 2020 than in 2021, which was related to the resumption of work and production. Meanwhile, it cannot be ignored that basic environmental protection measures have been continuously upgraded, such as prevention and regulations implemented by environmental protection departments, and the adjustment of the industrial structure and energy sources. Therefore, the effect of COVID-19 control measures on the AQI reduction should be smaller than that estimated by the simulations for each month.

The AQI can only reflect changes in the concentration of primary pollutants. From February to April 2020, the number of days in which PM_2.5_ or PM_10_ was the primary pollutant accounted for 70%, indicating that the AQI decrease was largely related to the decrease in particulate matter (PM) concentrations. In other words, restrictive measures implemented to combat the COVID-19 pandemic had a positive impact on reducing PM concentrations. The changes in the PM concentrations were mainly attributable to the reduction in anthropogenic emissions due to COVID-19 control measures, although seasonal influences might also have contributed in part [49].

### 3.3. Analysis of Factors Influencing Air Quality during the Spring Festival

#### 3.3.1. Interannual Differences in Air Quality

The related social survey showed that the level of anthropogenic activity during the Spring Festival in 2020 dropped significantly; the average daily indices of immigrant populations and emigrant populations decreased by 0.629 and 0.982, respectively, compared with the Spring Festival of 2019 (https://qianxi.baidu.com, accessed on 31 January 2023); and the level of congestion on highways decreased significantly compared with the Spring Festival in previous years (https://jiaotong.baidu.com, accessed on 31 January 2023).

However, during the Spring Festival in 2020, the average concentrations of PM_2.5_, PM_10_, SO_2_, NO_2_, CO, and O_3_-8h were 122.43 µg/m^3^, 158.29 µg/m^3^, 36.57 µg/m^3^, 50.29 µg/m^3^, 2.37 mg/m^3^, and 69.14 µg/m^3^, which were significantly higher than those recorded in 2019 and 2021. The average AQI was 156.86 during the Spring Festival in 2020, which was 95.03% and 32.61% higher than in 2019 and 2021, respectively. This short-term heavy pollution phenomenon is contrary to the trend of overall air quality improvement during the Level I response period and is not consistent with reduced anthropogenic activities, so it is worth investigating the reasons.

Due to the consistent control measures, the level of human activity and anthropogenic emissions were considered to be unchanged during the Level I response period, including the Spring Festival. Therefore, we suspect that some other factors offset the impact of anthropogenic emissions. In fact, similar phenomena were not rare during COVID-19 in other cities. Previous studies have shown that heavy pollution events often occurred in northern Chinese cities during COVID-19, which may be related to unfavorable meteorological conditions and regional transport (Table 2). Zhao et al. [50] pointed out that a large-scale air pollution event occurred on 23–28 January 2020, which led to PM_2.5_, SO_2_, NO_2_, and CO concentrations increasing in the Beijing–Tianjin–Hebei region. During this period, the Tangshan also suffered a heavy pollution event, and the Ecology and Environment issued a relevant notice (http://sthjj.tangshan.gov.cn/sthjj/xqdt/20200207/1334534.html, accessed on 31 January 2023). Therefore, the causes of heavy pollution events during the Spring Festival were analyzed from two aspects: meteorological conditions and regional transport.

#### 3.3.2. Impact of Meteorological Conditions

**Figure 6:**
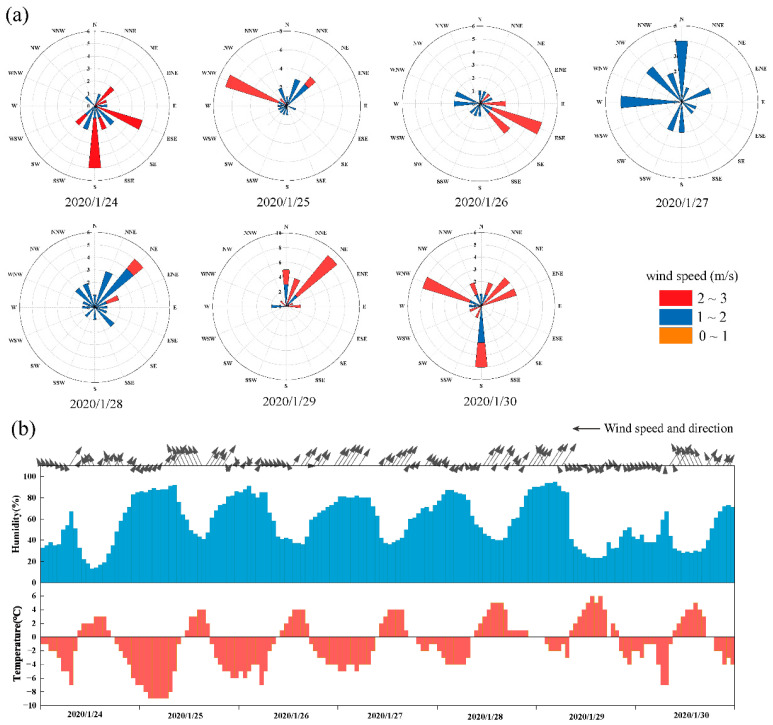
Daily and hourly changes in meteorological elements during the Spring Festival in 2020. (**a**) Daily wind rose diagram. (**b**) Hourly changes in temperature, humidity, wind speed, and direction (the direction of the arrow represents the wind direction, and the length of the arrow represents the wind speed).

The wind rose diagrams during the seven-day Spring Festival of 2020 are shown in Figure 6a, and the hourly changes in temperature, humidity, wind speed, and wind direction are illustrated in Figure 6b. The average temperature, humidity, and wind speed of these days were −1.03 °C, 57.85%, and 1.35 m/s, respectively (Supplementary Materials Table S4). The typical meteorological conditions that contributed to local heavy pollution events are wind speed less than 2 m/s, high static stability of atmosphere, temperature inversion near the ground, and a humidity of more than 60% [55]. When weak southerly winds prevailed on 24 January and 26 January, atmospheric pollutants were accumulated due to being blocked by the Yanshan–Taihang mountain chain [56]. The westerly and northerly winds predominantly prevailed on 27 January, but the wind speed was less than 2 m/s, which was an unfavorable meteorological condition. On 28 January, the northeast wind had a wind speed greater than 2 m/s, which allowed heavy pollution to begin to ease. In this study, temperature was negatively correlated with pollutants other than O_3_, and a cooling process was undergone from 24 January to 26 January, which may have led to an increase in particulate matter concentration and an increase in AQI. From January 24 pm to January 29 am, there were five peaks in the change in humidity, with an average humidity of 64.82%, and low-humidity (RH = 70–80%) conditions promoted the accumulation of particulate matter [57]. There was no precipitation during the Spring Festival, while the nights of 25 January, 26 January, 28 January, and 30 January were breezy, which favored the development of temperature inversions [58]. The average visibility during the Spring Festival in 2020 was 8.3 km, lower than in 2019 (22.56 km) and 2021 (13.93 km) (Supplementary Materials Table S4), which was also one of the signs of fog haze [59]. Therefore, the preliminary judgment showed that unfavorable meteorological conditions led to a large reduction in environmental capacity, and heavy pollution occurred even though the level of social activity was low.

#### 3.3.3. Impact of the Regional Transport

**Figure 7:**
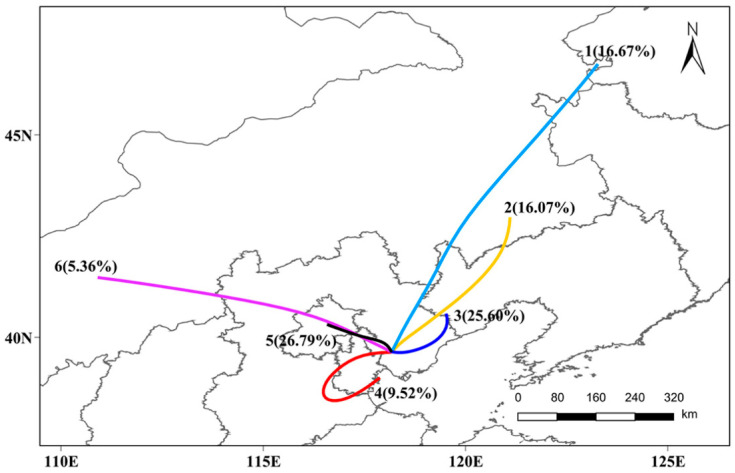
Backward trajectory clustering of air masses during the Spring Festival in 2020.

This section simulated the backward trajectory of air masses from 0:00 on 24 January to 23:00 on 30 January 2020, and identified possible transport routes for pollutants through cluster analysis. Through the total spatial variance analysis, all backward trajectories could be aggregated into six categories (Figure 7). The trajectory-3 from Qinhuangdao–Tangshan and the trajectory-5 from Beijing–Tianjin corresponded to air masses with a short-distance transport and accounted for 25.60% and 26.79% of the total trajectories, respectively. These air masses move slowly, which is not conducive to the diffusion of pollutants, and they are the most likely to carry the pollution to the receiving point. In contrast, the trajectory-1 from the northeastern region and the trajectory-6 from central Inner Mongolia corresponded to air masses with long-distance transport and accounted for 16.67% and 5.36% of the total trajectories, respectively. Due to their high speed and low possibility of occurrence, this type of air mass may have little effect on pollutant concentration in Tangshan.

**Figure 8:**
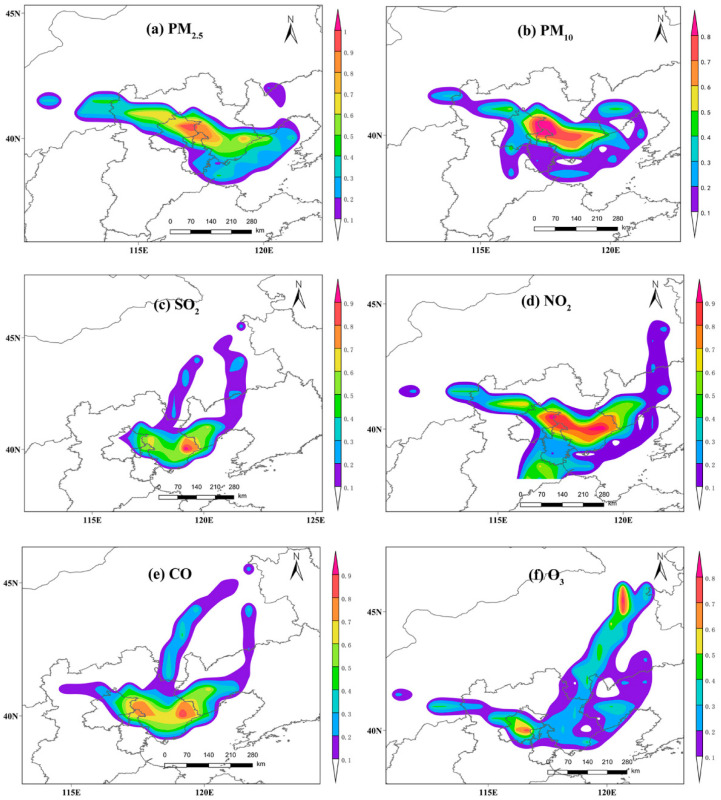
Analysis of potential areas acting as sources of atmospheric pollutants during the Spring Festival in 2020.

Based on the analysis of the transportation path, PSCF analysis was used to determine the main potential areas representing pollution sources (Figure 8). The results showed that local emissions and short-distance transportation were the main sources of air pollution in Tangshan. The main potential sources of PM_2.5_ and PM_10_ were located in the northern parts of Beijing and Tianjin, and this is specifically related to the increase in particulate matter concentration caused by the frequent occurrence of sandstorms in Beijing in winter and spring [60]. The main potential sources of SO_2_ and CO were located in Tangshan and Qinhuangdao, which were the leading cities in terms of growth rates of the industrial output value in Hebei Province in 2020, and the main sources of the two above-mentioned pollutants were the industries producing steel, energy, glass, and building materials. As an important indicator to evaluate the contribution of mobile sources, the potential sources of NO_2_ were mainly distributed between Beijing and Tangshan, suggesting that high-intensity anthropogenic activities may occur in these areas [61]. Affected by the northerly winter wind during the East Asian monsoon, O_3_ and its precursors from the cold inland part of northern China were transmitted to Tangshan through long-distance transportation [62].

## 4. Conclusions

From 24 January to 30 June 2020, Tangshan experienced the adjustment in the response level from Level I to Level II, and then to Level III, in which the corresponding control measures affected air quality. The comparative analysis and DID model were used to analyze the interannual differences in air quality after the implementation of COVID-19 control measures and the differences before and after COVID-19 in 2020, respectively. The MLR model successfully established a linear relationship between meteorological factors and AQI, which was used to quantitatively assess the contribution of COVID-19 control measures to air quality improvement during the Level I response. The Spring Festival was studied as a special period during the Level I response to explore possible sources of pollutants.

During COVID-19 in 2020, the average concentration of six conventional air pollutants decreased by 6.13–42.78% compared to 2017–2019, and the concentrations of PM_2.5_, PM_10_, and NO_2_ were lower than those recorded in 2021. Compared to the 23 days before the COVID-19 control measures began to be implemented, the measures did not result in a significant difference in air quality. Spearman correlation analysis showed that meteorological factors had a negative effect on air quality during COVID-19 in 2020. The analysis of the economic indicators showed that the decline in pollutant concentrations might be caused by the closure of industries unrelated to livelihood.

During the Level I response, air quality was significantly better in 2020 than in 2017–2019 and 2021. The COVID-19 control measures resulted in a significant difference in air quality at 95% confidence intervals for 23 days before and after the response was initiated. Under this assumption that the difference between the simulated and observed values are caused by COVID-19 control measures, the MLR model simulation results showed that the reduction in AQI caused by COVID-19 control measures were 29.07%, 31.43%, and 20.04% in February, March, and April of 2020, respectively. Furthermore, the stricter the COVID-19 control measures, the more obvious the air quality improvement effect.

During the Spring Festival period of 2020, the average concentrations of PM_2.5_, PM_10_, SO_2_, NO_2_, CO, and O_3_-8h were significantly higher than in 2019 and 2021. The simulated average value of AQI was 20.51% lower than the observed one. Unfavorable meteorological conditions and regional transport may offset the impact of anthropogenic emissions. Temperature, humidity, wind speed, and wind direction were all unfavorable meteorological factors. The analysis of the regional transport impact showed that local emissions and short-distance transport were the main sources of air pollution during the Spring Festival of 2020 in Tangshan.

In conclusion, strict COVID-19 control measures and meteorological factors have a more significant impact on the air quality over a time span of several months and several days, respectively. As for the continuous improvement in air quality, it is also necessary to adhere to the prevention and control of air pollution on the basis of paying attention to the impact of meteorology.

## Figures and Tables

**Table 1:** Main economic indicators for the city of Tangshan in the first and second quarters of the 2019–2021 period.

Economic Indicator	Unit	2019		2020		2021	
		1st	2nd	1st	2nd	1st	2nd
*GDP of the Secondary Industry*	Billion Yuan	82.21	188.79	73.69	171.49	91.91	208.74
*Total Profit of Manufacturing*	Billion Yuan	3.79	16.46	1.57	8.35	/	/
*Product Output*							
Steel	Million Tons	34.12	40.41	34.74	44.46	38.74	37.53
Coal	Million Tons	5.98	5.73	5.60	5.47	5.07	4.80
Cement	Million Tons	3.92	9.05	3.42	10.88	5.64	10.90
Power Generation	Billion kWh	16.6	15.6	16.8	16.9	19.7	16.4
*Electricity Consumption*							
Industry	Billion kWh	16.72	18.08	15.18	18.18	17.26	18.29
Construction	Billion kWh	0.19	0.13	0.16	0.14	0.23	0.17
Urban and Rural Life	Billion kWh	1.36	1.08	1.52	1.14	1.75	1.21
*Operating Income of Transportation, Warehousing and Postal Industry*	Billion Yuan	8.52	13.22	7.77	15.18	8.51	14.15

**Table 2:** Summary of studies related to the causes of air pollution events during COVID-19.

Study	Region	Phenomenon	Reason
[19]	Beijing–Tianjin–Hebei (BTH)	Persistent high PM_2.5_ pollution.	Unfavorable meteorological conditions.
[50]	BTH	Two large-scale air pollution events led to higher concentrations of PM_2.5_, SO_2_, NO_2_, and CO.	Regional transport and unfavorable meteorological conditions.
[51]	Beijing, Tianjin, and Baoding	Heavy haze pollution.	Inter-transport of PM_2.5_ in the BTH region and unfavorable meteorological conditions.
[52]	BTH	Higher aerosol and PM_2.5_ levels in February and March 2020.	Enhanced atmospheric oxidation capacity.
[47]	Eastern China	The concentration of PM_2.5_ increased during the COVID-19 lockdown.	Enhanced secondary pollution offset reduction in primary emissions.
[53]	Baoding	A heavy pollution event.	Regional transport and unfavorable meteorological conditions.
[54]	Beijing	Heavy PM_2.5_ pollution.	The initial regional transport and later secondary formation under adverse meteorology.
[54]	Henan	Heavy PM_2.5_ pollution.	The primary emissions and small-scale regional transport.

## Data Availability

The data used to support the findings of this study are available from the corresponding author upon request.

## Supplementary Materials

The following supporting information can be downloaded at: https://www.mdpi.com/article/10.3390/ijerph20054199/s1, Table S1: Average values and interannual differences of AQI and concentrations of six air pollutants in different periods of 2017–2021.; Table S2A: Descriptive statistics of the main variable.; Table S3A: Parameters estimation of DID.; Figure S1A: The parallel trend test results of DID analyses.; Table S2B: Descriptive statistics of the main variable.; Table S3B: Parameters estimation of DID.; Figure S1B: The parallel trend test results of DID analyses.; Table S4: Average values of six meteorological parameters in different periods of 2017–2021.; Figure S2: Emission contribution rate of pollution sources based on the Air Pollution Source Emission Inventory of Tangshan (2017).; Text S1. Comparative analysis of MLR model and PCR model.; Table S5. The MLR models information.; Figure S3. P–P (normal probability) plots of standardized residuals in the MLR.; Table S6. The PCR models information.; Figure S4. P–P (normal probability) plots of standardized residuals in the PCR.; Table S7. The regression equations of the MLR models.
