# Supplementary material for: Analysis of Factors Influencing Air Quality in Different Periods during COVID-19: A Case Study of Tangshan, China
Source: Int J Environ Res Public Health. 2023 Feb 26;20(5):4199. doi: 10.3390/ijerph20054199 (PMC10002059; doi:10.3390/ijerph20054199)
Supplement: Supplementary file 1 [file ijerph-20-04199-s001.zip › ijerph-2225134-supplementary.pdf]

Supporting Information for

Analysis of factors influencing air quality in different periods during COVID-19: A case study of Tangshan, China

Wenlu Wu, Chunyan Shan*, Jing Liu, Jinglin Zhao, Jinyun Long

College of Environmental Science and Engineering, Nankai University, Tianjin, 300350, People's Republic of China

Corresponding author: Chunyan Shan

E-mail: shanchy@nankai.edu.cn

Present address: College of Environmental Science and Engineering, Nankai University, No.38 Tongyan Road, Jinnan District, Tianjin 300350, PR China

* Corresponding author, Email: shanchy@nankai.edu.cn

Table S1. Average values and interannual differences of AQI and concentrations of six air pollutants in different periods of 2017–2021.

Index	Period	Average value			Difference between	Difference between
		2017–2019	2020	2021	2017–2019 & 2020（%）	2020 & 2021（%）
AQI	Jan. 24 to Jun. 30	110.35	93.20	101.23	-15.54***	8.62
	Jan. 24 to Apr. 30	105.72	88.76	108.09	-16.04***	21.78**
$PM_{2.5}$ (μg/m^3)	Jan. 24 to Jun. 30	62.91	50.03	54.33	-20.47**	8.59
	Jan. 24 to Apr. 30	71.40	56.22	67.22	-21.26***	19.57*
PM_{10} (μg/m^3)	Jan. 24 to Jun. 30	122.07	93.04	114.03	-23.78***	22.56**
	Jan. 24 to Apr. 30	128.52	96.93	134.60	-24.58***	38.86***
SO_2 (μg/m^3)	Jan. 24 to Jun. 30	34.71	19.86	12.16	-42.78***	-38.77***
	Jan. 24 to Apr. 30	35.90	19.38	14.69	-46.02***	-24.20**
NO_2 (μg/m^3)	Jan. 24 to Jun. 30	53.43	41.36	41.78	-22.59***	1.02
	Jan. 24 to Apr. 30	56.45	41.73	49.82	-26.08***	19.39**
CO (mg/m^3)	Jan. 24 to Jun. 30	1.67	1.25	1.02	-25.15***	-18.40**
	Jan. 24 to Apr. 30	1.73	1.32	1.19	-23.70***	-9.85
O_3-8h (μg/m^3)	Jan. 24 to Jun. 30	117.36	110.17	100.32	-6.13	-8.94*
	Jan. 24 to Apr. 30	88.28	87.80	80.74	-0.54	8.04

*** p<0.01, ** p<0.05, * p<0.1

Index	Period	Average value			Difference between	Difference between
		2019	2020	2021	2019 & 2020（%）	2020 & 2021（%）
AQI	the Spring Festival	80.43	156.86	118.29	95.03*	-24.59
$PM_{2.5}$ (μg/m^3)	the Spring Festival	52.57	122.43	84.71	132.89*	-30.81
PM_{10} (μg/m^3)	the Spring Festival	105.14	158.29	114.29	50.55	-27.80
SO_2 (μg/m^3)	the Spring Festival	24.00	36.57	17.29	52.38	-52.72**
NO_2 (μg/m^3)	the Spring Festival	29.71	50.29	33.29	69.27**	-33.80
CO (mg/m^3)	the Spring Festival	1.44	2.37	1.63	64.58*	-31.22
O_3-8h (μg/m^3)	the Spring Festival	59.43	69.14	66.57	16.34	-3.72

*** p<0.01, ** p<0.05, * p<0.1

Difference-in-differences (DID) model I.

Table S2A. Descriptive statistics of the main variable.

Variable	Unit	Obs	Mean	Std. Dev.	Min	Max
lnAQI	Index	847	4.59	0.48	3.33	6.21
$lnAQI_{t-1}$	Index	847	4.58	0.48	3.33	6.21
Temperature	℃	847	9.79	11.06	-15.5	30.9
Humidity	%	847	52.19	16.63	17	97
Wind speed	m/s	847	2.55	1.07	0.1	8.2
Visibility	km	847	18.88	8.55	0.9	30
Precipitation	mm	847	0.78	5.43	0	107.1

Table S3A. Parameters estimation of DID.

VARIABLES	lnAQI
$lnAQI_{t-1}$	0.386***
	(0.0249)
Temperature	0.00825***
	(0.00106)
Humidity	-0.00417***
	(0.000995)
Wind speed	-0.0371***
	(0.0122)
Visibility	-0.0321***
	(0.00209)
Precipitation	-0.0125***
	(0.00208)
Trend	-0.0608***
	(0.0131)
Measure*Post	-0.142***
	(0.0376)
Constant	3.840***
	(0.163)
Observations	847
R-squared	0.575

Standard errors in parentheses
*** p<0.01, ** p<0.05, * p<0.1

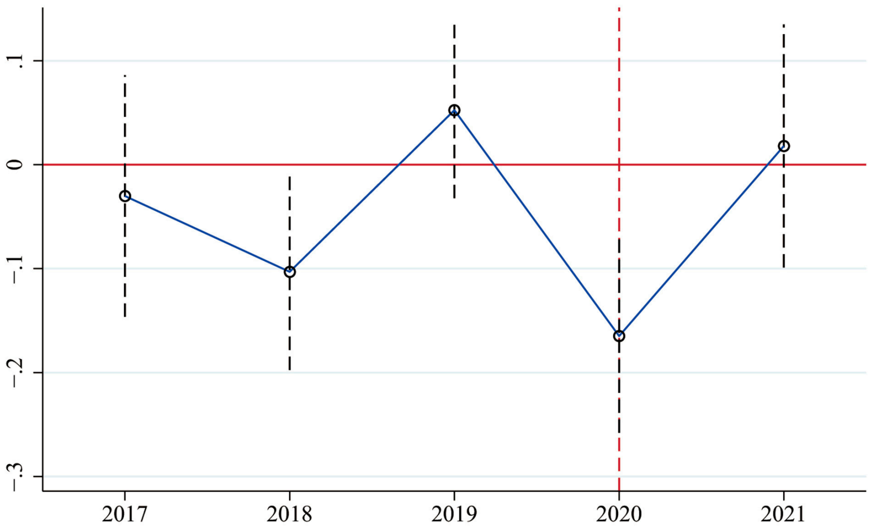

Figure S1A. The parallel trend test results of DID analyses.

Difference-in-differences (DID) model II.

Table S2B. Descriptive statistics of the main variable.

Variable	Unit	Obs	Mean	Std. Dev.	Min	Max
lnAQI	Index	230	4.66	0.55	3.33	6.00
$lnAQI_{t-1}$	Index	230	4.66	0.55	3.33	6.00
Temperature	°C	230	-3.59	3.42	-15.5	6.6
Humidity	%	230	53.23	18.31	17	97
Wind speed	m/s	230	2.06	1.16	0.1	8.2
Visibility	km	230	16.65	9.18	0.9	30
Precipitation	mm	230	0.12	0.90	0	11.7

Table S3B. Parameters estimation of DID.

VARIABLES	lnAQI
$lnAQI_{t-1}$	0.386***
	(0.0451)
Temperature	0.00920
	(0.00751)
Humidity	0.00262
	(0.00185)
Wind speed	-0.0477**
	(0.0236)
Visibility	-0.0261***
	(0.00397)
Precipitation	-0.0525**
	(0.0258)
Measure*Post	-0.317***
	(0.0771)
Constant	3.323***
	(0.271)
Observations	230
R-squared	0.661

Standard errors in parentheses
*** p<0.01, ** p<0.05, * p<0.1

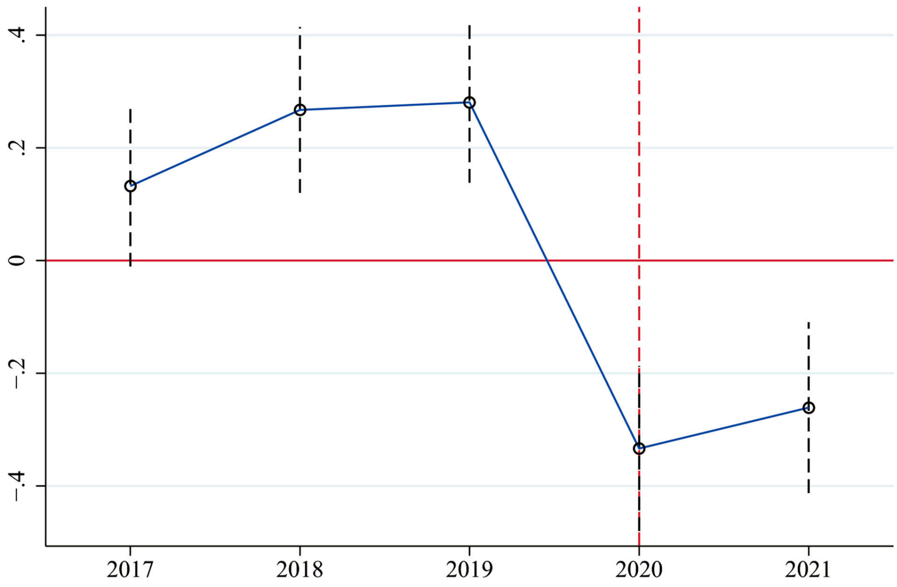

Figure S1B. The parallel trend test results of DID analyses.

Table S4. Average values of six meteorological parameters in different periods of 2017–2021.

period		Temperature (℃)	Humidity (%)	Wind speed (m/s)	Atmospheric pressure (hPa)	Visibility (km)	Precipitation (mm)
Jan. 24 to Jun. 30	**2017**	12.96	51.29	2.79	1013.	24.60	0.35
	**2018**	12.30	51.05	2.91	1013	19.76	0.60
	**2019**	12.28	47.48	2.13	1013	20.41	0.64
	**2020**	10.56	54.30	1.92	1015	16.09	0.73
	**2021**	12.17	54.56	2.82	1012	15.64	1.82
the Spring Festival	**2019**	-4.92	36.01	1.85	1026	22.56	0
	**2020**	-1.03	57.86	1.35	1027	8.3	0
	**2021**	1.14	62.71	1.57	1019	13.93	0

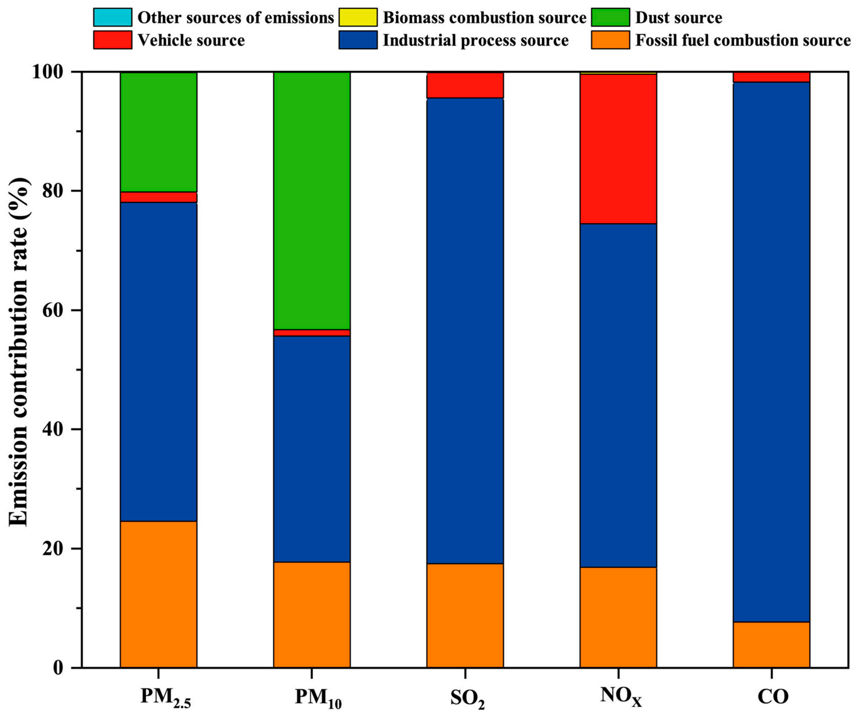

Figure S2. Emission contribution rate of pollution sources based on the Air Pollution Source Emission Inventory of Tangshan (2017).

According to the Air Pollution Source Emission Inventory of Tangshan (2017), industrial processes, fossil fuel combustion, and dust were the main sources of $PM_{2.5}$, with the emission contribution rates were 53.55%, 24.61%, and 20.09%, respectively; dust, industrial processes, and fossil fuel combustion were the main sources of PM_{10}, with the emission contribution rates were 43.21%, 38.03%, and 17.70%, respectively; industrial processes, fossil fuel combustion, and vehicle were the main sources of SO_2, with the emission contribution rates were 78.16%, 17.42%, and 4.31%, respectively; industrial processes, fossil fuel combustion, and vehicle were the main sources of CO, with the emission contribution rates were 90.68%, 7.57%, and 1.70%, respectively; industrial processes, vehicle source, and fossil fuel combustion were the main sources of NOx, with the emission contribution rates were 57.64%, 25.13%, and 16.84%, respectively.

Text S1. Comparative analysis of MLR model and PCR model.

Multiple linear regression (MLR) and principal component regression (PCR) are common machine learning methods used to predict air quality. MLR or PCR model can be established to achieve the purpose of prediction based on the data and causal relationship of air quality and meteorological elements.

In this study, MLR model and PCR model were tested to simulate the relationship between six meteorological parameters and the air quality index (AQI) for February, March and April in 2017–2019, respectively. Residual plots are used to validate assumptions about the regression model. The coefficient of determination R^2, R^2 -adjusted, and mean absolute percentage error (MAPE) were used to evaluate the quality of the model; the calculation formulas are as follows:

$$R^2 = \left[\frac{\sum_{i=1}^{n}(O_i - \bar{O})^2 \cdot (P_i - \bar{P})}{n \cdot \sigma_o \cdot \sigma_p}\right]^2 \tag{S1}$$

$$MAPE = \frac{1}{n}\sum_{i=1}^{n}\frac{|P_i - O_i|}{O_i} \tag{S2}$$

where O_i represents the observed value, and P_i represents the predicted value.

The independent variables include *temperature*, *humidity*, *wind speed*, *atmospheric pressure*, *visibility*, and *precipitation*. The dependent variable is *logAQI*.

The results were shown in Table S5, Figure S3, Table S6, Figure S4. The P-value < 0.05 indicates that the establishment of MLR models or PCR models is reasonable. However, from the three aspects of *R^2-adjusted*, MAPE, and P–P (normal probability) plots, the MLR model was more suitable for this study. The regression equations of the MLR models were shown in Table S7.

Table S5. The MLR models information.

Month	Index	Method	Residual distribution	F	P-value	R^2 -adjusted	MAPE
February	AQI	MLR	Normality	54.895	0.000	0.798	16.76%
March	AQI	MLR	Normality	53.933	0.000	0.702	19.99%
April	AQI	MLR	Normality	21.131	0.000	0.599	21.86%

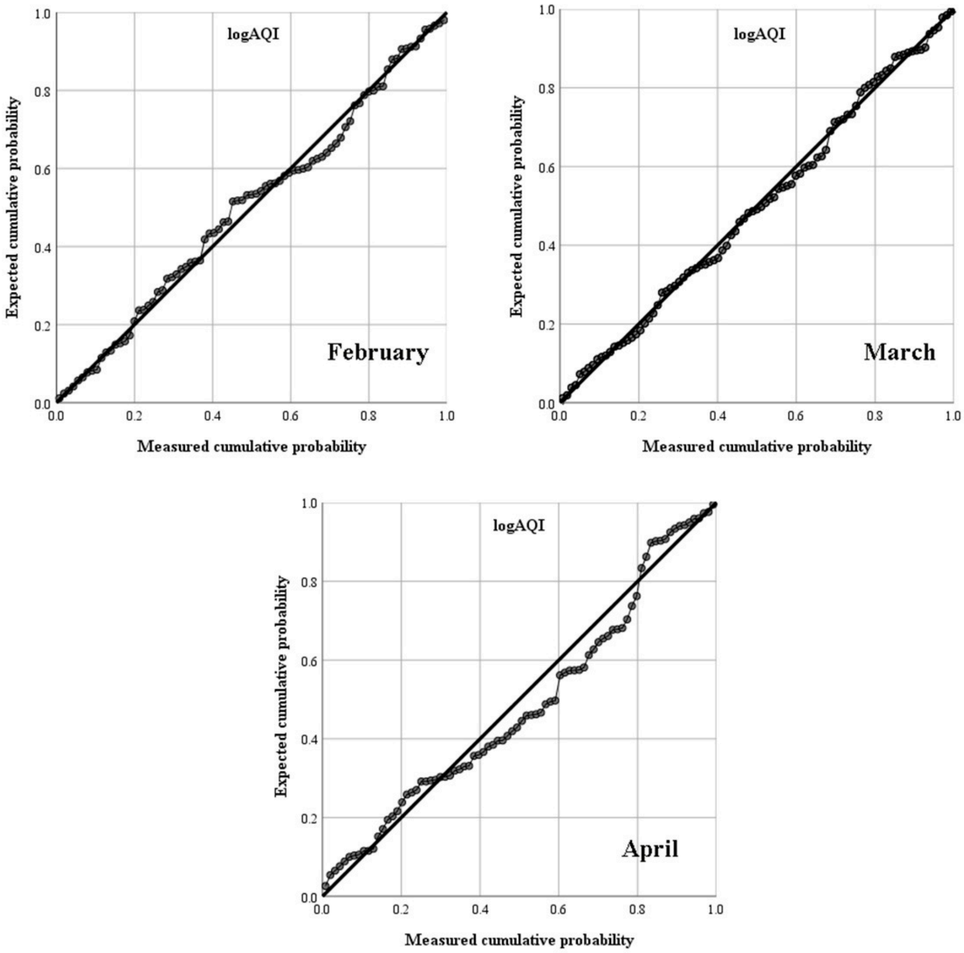

Figure S3. P–P (normal probability) plots of standardized residuals in the MLR.

Table S6. The PCR models information.

Month	Index	Method	Residual distribution	F	P-value	R^2 -adjusted	MAPE
February	AQI	PCR	Normality	155.458	0.000	0.790	18.69%
March	AQI	PCR	Normality	45.476	0.000	0.597	23.45%
April	AQI	PCR	/	/	/	/	/

/: The KMO < 0.5 in April, which means the model is not suitable.

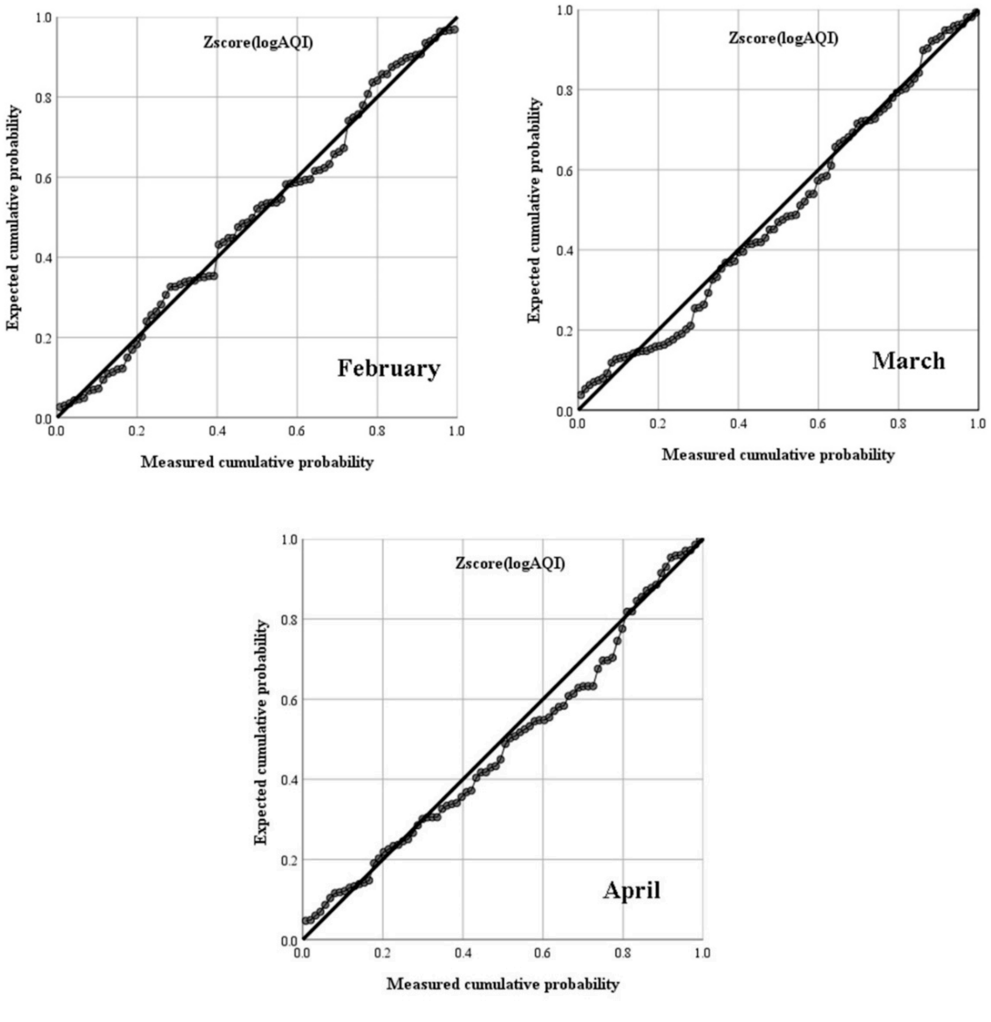

Figure S4. P–P (normal probability) plots of standardized residuals in the PCR.

Table S7. The regression equations of the MLR models.

Month	Regression equation
February	$log_{AQI} = 9.093 + 0.008x_1 + 0.005x_2 + 0.001x_3 - 0.007x_4 - 0.013x_5 - 0.046x_6$
March	$log_{AQI} = 1.868 + 0.012x_1 + 0.006x_2 - 0.0012x_4 - 0.112x_6$
April	$log_{AQI} = 9.334 + 0.004x_1 - 0.002x_2 + 0.005x_3 - 0.007x_4 - 0.011x_5 - 0.0189x_6$

x_1: temperature; x_2: humidity; x_3: wind speed; x_4: atmospheric pressure, x_5: visibility; x_6: precipitation.
